# Supplementary material for: Gelatin methacrylate hydrogels culture model for glioblastoma cells enriches for mesenchymal-like state and models interactions with immune cells
Source: Sci Rep. 2021 Sep 6;11:17727. doi: 10.1038/s41598-021-97059-z (PMC8421368; doi:10.1038/s41598-021-97059-z)
Supplement: Supplementary file 1 — Supplementary Information 1. [file 41598_2021_97059_MOESM1_ESM.docx]

**Gelatin methacrylate hydrogels culture model for glioblastoma cells enriches for mesenchymal-like state and models interactions with immune cells**

**Supplemental Information:**

**Supplementary Figure legends:**

**Supplementary Figure-S1: Phenotypic properties of glioma cells.**

Bright-field microscopy images depicting the morphology of MN478 (patient-derived glioma cells) grown in 2D, and 3D-GMH.

** Supplementary Figure-S2: Glioma cells cultured as neurospheres show increased invasive property**

Quantification of Boyden chamber invasion assay for U251 cells and MN238 patient-derived glioma cells cultured in 2D and as GSC.

**
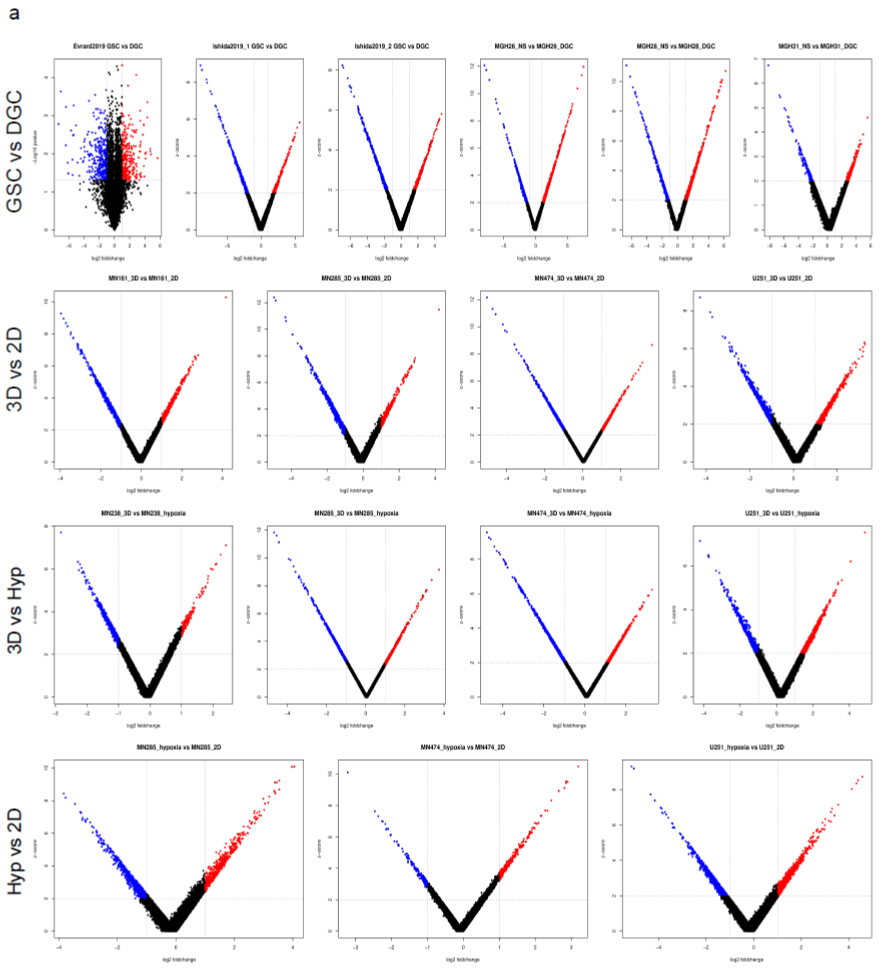
 Supplementary Figure-S3:** **Volcano plots representing differentially expressed genes across 14 comparisons, 3D-GMH, GSC and mechanosensing genesets**

Volcano plots for four comparisons namely, GSC vs. DGC, 3D-GMH vs. 2D, 3D-GMH vs. hypoxia, and hypoxia vs. 2D. For the Evrard microarray dataset -log10 of p-value and log2 fold change values were used for plotting the volcano plot. For all the other datasets, the single sample comparison method was used for the identification of differentially expressed genes. As the p-value could not be calculated for the same, an absolute z-score value was used along the y-axis with log2 fold change values on the x-axis. Each dot represents a gene. Any gene with z-score >2 and log2 fold change >1 was considered to be upregulated and z-score >2 and fold change <-1 was considered to be downregulated. The red dots represent upregulated genes whereas the blue dots represent the downregulated genes.

** Supplementary Figure-S4:** **Glioma cells cultured in 3D-GMH secrete enhanced cytokine and chemokines** (A) Cytokine and chemokine expression levels in MN474 glioma cells cultured as GSC (B) Relative fold change in cytokine/chemokine expression in GSC, mean pixel density is normalized to 2D. (C) Cytokine and chemokine expression levels in MN478 glioma cells cultured in 3D-GMH (Top) and in 2D (Bottom) Magenta/pink bordered cytokine/chemokine indicates increased secretion in cells cultured in 3D-GMH and Cyan bordered cytokine/chemokine indicates increased secretion in cells cultured in 2D. (D) Relative fold change in cytokine/chemokine expression in 3D-GMH, mean pixel density is normalized to 2D.

** Supplementary Figure-S5: Glioma cells cultured in 3D-GMH enhances primary monocyte recruitment** (A) Boyden chamber invasion assay for U937-derived macrophages against conditioned media of U251 cells and patient-derived glioma cells cultured in 2D and GSC (B) Boyden chamber invasion assay for primary human monocytes against conditioned media of MN238 patient-derived glioma cells cultured in 2D, 3D-GMH and as GSC. (C) Representative images of primary human monocytes attracted towards conditioned media of MN238 patient-derived glioma cells cultured in 2D, 3D-GMH and as GSC.

**Supplementary Files:**

1. **Supplementary File -1:** Representative patient-derived glioma and glioblastoma cell lines cultured under different conditions.
2. **Supplementary File -2:** Gene matrix indicating the differential genes across 14 comparisons 3D-GMH genelist, GSC genelist, and mechanosensing genelist.
3. **Supplementary File -3:** Neftel meta-modules genesets used for SSGSEA
4. **Supplementary File -4:** TCGA and CCGA samples survival data
