# Supplementary material for: Gelatin methacrylate hydrogels culture model for glioblastoma cells enriches for mesenchymal-like state and models interactions with immune cells
Source: Sci Rep. 2021 Sep 6;11:17727. doi: 10.1038/s41598-021-97059-z (PMC8421368; doi:10.1038/s41598-021-97059-z)
Supplement: Supplementary file 2 — Supplementary Information 2. [file 41598_2021_97059_MOESM2_ESM.docx]

**Table 1:** Representative patient-derived glioma and GBM cell lines cultured under different conditions.

| **Description** | **Cell line** | **Patient-derived glioma cells** | | | | | | |
| --- | --- | --- | --- | --- | --- | --- | --- | --- |
| MN NO | U251 | MN238 | MN161* | MN407 | MN298* | MN474 | MN285 | MN478 |
| Pathology | Glioblastoma | Glioblastoma | Recurrent glioblastoma | Glioblastoma | Glioblastoma | Glioblastoma | Anaplastic astrocytoma | Glioblastoma |
| IDH1 status |  | IDH1 WT | IDH1 Mutant R132H | IDH1 WT | IDH1 Mutant not R132H | IDH1 WT | IDH1  WT | IDH1 WT |
| Passage |  | P11 | P1 | P1 | P3 | P1 | P4 | P1 |
| 2D |  | 🗸 | 🗸 | 🗸 | 🗸 | 🗸 | 🗸 | 🗸 |
| 2D hypoxia 48hours | 🗸 | 🗸 |  | 🗸 | 🗸 | 🗸 | 🗸 | 🗸 |
| Neurospheres | 🗸 | 🗸 |  |  |  |  |  |  |
| 3D 15 days | 🗸 | 🗸 | 🗸 | 🗸 | 🗸 | 🗸 | 🗸 | 🗸 |
| Chemoresponse | 🗸 | 🗸 |  |  | 🗸 | 🗸 |  |  |
| Invasion | 🗸 | 🗸 |  |  | 🗸 | 🗸 |  |  |
| Macrophage infiltration | 🗸 | 🗸 |  | 🗸 |  | 🗸 |  | 🗸 |
| Cytokine array |  |  |  |  |  | 🗸 |  | 🗸 |
| Cell cycle analysis | 🗸 |  |  |  |  |  |  |  |

(* = IDH1 mutations were found to be lost in the cultures)
